# Improvement of Motor Task Performance: Effects of Verbal Encouragement and Music—Key Results from a Randomized Crossover Study with Electromyographic Data

**DOI:** 10.3390/sports12080210

**Published:** 2024-07-30

**Authors:** Filippo Cotellessa, Nicola Luigi Bragazzi, Carlo Trompetto, Lucio Marinelli, Laura Mori, Emanuela Faelli, Cristina Schenone, Halil İbrahim Ceylan, Carlo Biz, Pietro Ruggieri, Luca Puce

**Affiliations:** 1 Department of Neuroscience, Rehabilitation, Ophthalmology, Genetics, Maternal and Child Health, University of Genoa, 16132 Genoa, Italy; filippo_cotellessa@hotmail.it (F.C.); ctrompetto@neurologia.unige.it (C.T.); lucio.marinelli@unige.it (L.M.); morilaurab@gmail.com (L.M.); crischenone92@gmail.com (C.S.); 2 Department of Food and Drugs, University of Parma, 43125 Parma, Italy; robertobragazzi@gmail.com; 3 IRCCS Ospedale Policlinico San Martino, 16132 Genoa, Italy; 4 Department of Experimental Medicine, Section of Human Physiology, University of Genoa, 16132 Genoa, Italy; emanuela.faelli@unige.it; 5 Department of Physical Education of Sports Teaching, Faculty of Sports Sciences, Atatürk University, Erzurum 25240, Türkiye; halil.ibrahimceylan60@gmail.com; 6 Orthopedics and Orthopedic Oncology, Department of Surgery, Oncology and Gastroenterology (DiSCOG), University of Padua, 35128 Padua, Italy; carlo.biz@unipd.it (C.B.); pietro.ruggieri@unipd.it (P.R.)

**Keywords:** motor recruitment, muscle excitation, muscle fatigue, endurance task, motivation

## Abstract

External motivational stimuli have been shown to improve athletic performance. However, the neurophysiological mechanisms underlying this improvement remain poorly understood. This randomized crossover study investigated the effects of music and verbal encouragement on measures of muscle excitation and myoelectric manifestations of fatigue in the biceps brachii and brachioradialis muscles during an endurance task. Fifteen untrained (mean age 29.57 ± 2.77 years) and 13 trained individuals (mean age 32.92 ± 2.90 years) were included. The endurance task, performed to exhaustion, consisted of keeping the dominant arm flexed to 90 degrees while holding a dumbbell loaded to 80% of 1RM with a supine grip in three randomized conditions: standard, with self-selected music, and with verbal encouragement. The untrained subjects showed an increase in task duration of 15.26% (*p* < 0.003) with music and 15.85% (*p* < 0.002) with verbal encouragement compared to the condition without external stimuli. There were no significant differences in the myoelectric manifestations of fatigue between the different conditions. Regarding the muscle excitation metrics, although the mean amplitude, peak value, and area under the curve remained unchanged across conditions, a significant reduction in the trend coefficient, indicating motor unit recruitment over time, was observed with both music (biceps brachii: −10.39%, *p* < 0.001; brachioradialis: −9.40%, *p* < 0.001) and verbal encouragement (biceps brachii: −7.61%, *p* < 0.001; brachioradialis: −6.51%, *p* < 0.001) compared to the standard condition. For the trained participants, no significant differences were observed between conditions in terms of task duration and outcome measures related to muscle excitation and myoelectric manifestations of fatigue, suggesting the possible presence of a ceiling effect on motivation. These results highlight the important role of external motivational stimuli, such as music and verbal encouragement, in improving task performance in untrained subjects, probably through more effective and efficient recruitment of motor units.

## 1. Introduction

Verbal encouragement (VE) and the use of music have been extensively studied for their potential to improve physical performance through a variety of mechanisms [1,2]. With regard to VE, one possible mechanism underlying its effectiveness is the *startle mechanism*, a defensive reflex located in the brainstem that is activated in the presence of intense acoustic stimuli such as VE [3]. This reflex is thought to enhance neural drive leading to increased force through several mechanisms: increasing motor unit recruitment, intensifying motor unit firing frequency, suppressing supraspinal inhibition, and regulating psychomotor arousal [4,5,6]. In addition, VE was found to facilitate optimal mind–body alignment and help overcome challenges [7]. In this context, Bandura’s concept of self-efficacy plays a key role, highlighting how confidence in one’s abilities is crucial for optimal performance [8].

VE is effective in improving athletic performance across a wide range of disciplines, both on land [9,10] and in water [11], showing improvements not only in applied strength and endurance during dynamic activities such as soccer, tennis, cycling, running, and swimming [2,7,11,12] but also in the effectiveness of isometric and concentric muscle contractions in a variety of experimental contexts [4,13,14]. This approach has led to significant increases not only in motor performance but also in physiological outcomes, including increases in heart rate and maximal oxygen consumption [15,16].

However, recent studies suggest that in experienced athletes, the effectiveness of VE may be limited or even counterproductive by interfering with attentional focus strategies. For example, only modest improvements in upper body performance have been observed in elite rugby players [17], just as there was no increase in isometric grip strength in senior male judokas [18]. Similarly, comparing CrossFit sessions performed with and without VE in experienced athletes showed only modest improvements in strength performance when encouragement was present, whereas no significant difference was observed between the two conditions for high-intensity endurance exercises [19].

Similarly, with regard to music, several studies have shown that it can be very useful for improving performance, especially if fast and loud [20]. The main mechanism of this effect would be to divert concentration from feelings of fatigue during exercise [21]. Furthermore, music can modify psychomotor arousal acting as both a stimulant and a sedative before and during physical activity, depending on the needs of the athlete and the context [22]. Further research has shown that synchronizing the tempo of the music with the movement of the performer not only improves motor efficiency but can also optimize energy expenditure (kinetic and/or physiological efficiency) [23,24]. The efficacy of music in enhancing performance is so pronounced that its use has been banned in competitive sports settings.

Scientific research dealing with the effects of music on exercise performance has traditionally focused on examining a variety of physiological and perceptual parameters. These include task time, exercise endurance, lactate levels, maximal oxygen consumption, and the subjective experience of exertion [25,26,27,28,29].

However, there is a lack of neurophysiological research using surface electromyography (EMG) that examines the precise effects of both VE and music on muscle excitation and the performer’s ability to resist muscular fatigue, suggesting that this is an under-explored area that could yield important insights. Moreover, a recent multi-level meta-analysis of 139 studies to quantify the effects of listening to music during exercise and sport found a paucity of studies that compared trained and untrained participants in a laboratory setting [30]. Finally, there is a dramatic dearth of data concerning a direct comparison between the effects of VE and musical listening. To our knowledge, only one study [31] has examined their effects on agility tests of young basketball players and found no significant differences in performance times.

Based on these premises, the aim of the present study was to investigate, in a group of subjects with different levels of physical fitness, muscle excitation and myoelectric manifestations of fatigue using EMG, as well as task duration, during an isometric exhaustion task, performed in three different conditions: standard conditions, when listening to self-selected motivational music, and with VE. In the absence of previous specific studies, muscle excitation and fatigue were considered as exploratory variables, with no pre-defined hypotheses. On the contrary, based on the pertinent literature, VE and musical conditions were expected to improve performance, particularly in untrained participants.

## 2. Materials and Methods

### 2.1. Study Design and Ethics Approval

This study used a single-blind randomized crossover methodological approach to analyze the effect of three different environmental interventions—i.e., standard conditions, music, and VE—on performance in an isometric endurance task, comparing results between untrained and trained individuals. The intervals between each intervention, that is to say, the wash-out, were set at 10 days. The examinations and analyses were performed at the University Hospital “IRCCS Ospedale Policlinico San Martino”, Genoa, Italy. The study protocol was reviewed and approved by the local ethics committee of the University of Genoa, Genoa, Italy (protocol number 2024.37, 12 April 2024) and registered on the public website ClinicalTrials.gov (identifier: NCT06408155). Before the beginning of the experimental protocol, all participants were informed that they would undergo three different tests. However, the specific methods were not disclosed to them. They provided written, informed consent to participate in the study. Both the evaluators and the statistician were blinded to the details.

### 2.2. Inclusion and Exclusion Criteria

To be included in the study, candidates had to be in good general health, free of medical conditions that could interfere with their ability to perform the test safely and effectively. In addition, to be considered trained, participants had to perform at least 150 min per week of moderate-intensity endurance physical activity, equivalent to ≈1200 METs (metabolic equivalent of task) per minute. They were also required to perform at least 75 min per week of muscle-strengthening activities, equivalent to ≈300 METs per minute [32]. To accurately estimate the participants’ weekly MET minutes, a questionnaire was administered to collect information on the duration and intensity of each activity performed by the participants [33]. Participants who did not meet these physical activity standards were considered untrained. Exclusion criteria included a history of surgery to the arm, shoulder, or nearby areas that might limit the ability to perform the test. The use of medications that affect muscle function or pain perception was also a reason for exclusion, so that the test results could not be influenced by external factors.

### 2.3. Sample Size Calculation

An a priori sample size and power analysis was carried out using the open-source G*Power (version 3.1.9.7). Assuming an alpha error probability of 0.05 and 80% power, with two groups, a variable consisting of three levels, and an effect size f of 0.40, the computation yielded an overall sample size of at least 14 per group [34].

### 2.4. Isometric Exhaustion Task

**Figure 1:**
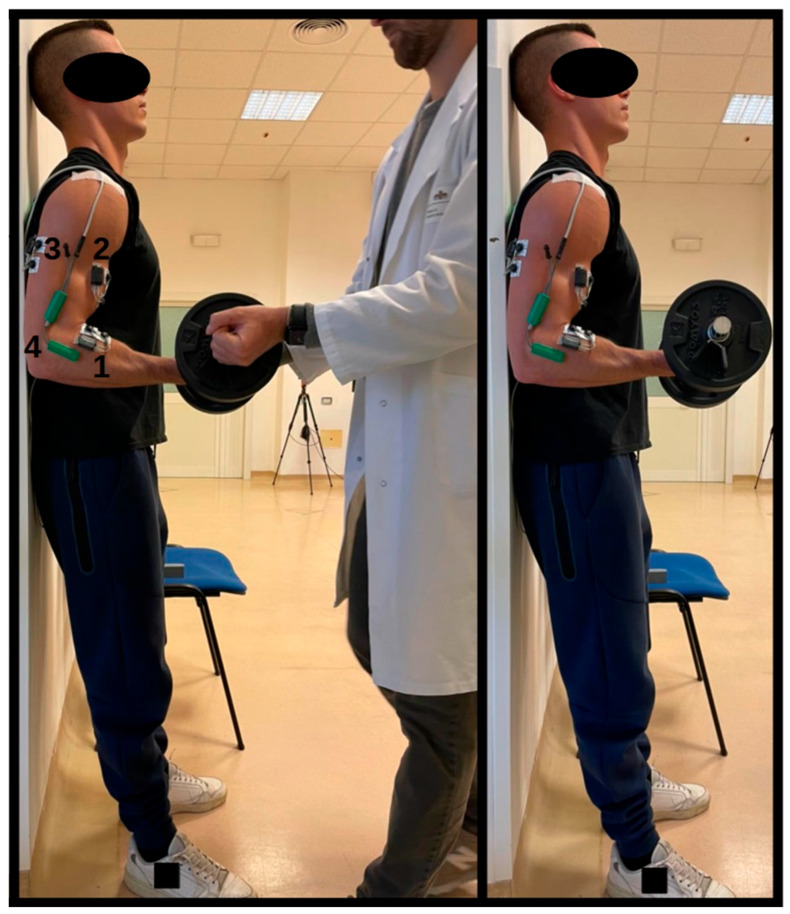
Position during the task. The task began when the operator released the weight into the subject’s hands. The test was completed when the subject could no longer keep their elbow bent at 90 degrees. 1 = brachioradialis EMG probe; 2 = biceps brachii EMG probe; 3 = triceps brachii EMG probe; 4 = electrogoniometer.

The protocol for each experimental task initially included a warm-up consisting of three sets of eight repetitions each, using a load equivalent to 50% of the individual’s one-repetition maximum (1RM), with a 2 min rest between each set. Next, the main exercise required participants to keep their arm flexed at a 90° angle while holding a dumbbell with a supine grip, loaded to 80% of their 1RM, on the dominant side of the body. During execution, the back and head were kept in contact with a vertical wall, with the feet shoulder width apart and firmly planted on the floor. The dumbbell was held with the dominant arm while the opposite arm remained in a neutral position close to the body (Figure 1). It was mandatory to avoid any form of rocking, swaying, or movement that might facilitate holding the position, thus ensuring the integrity of the muscular endurance test. The test officially began when the dumbbell was handed to the subject, who was already in the correct position, and ended as soon as the angle of the arm varied by more than 5 degrees, both in flexion and in extension. The angle of the elbow joint was continuously monitored with an electronic goniometer (model TDS130B, Biopac System Inc, Goleta, CA, USA) placed directly on the joint. A trained operator would interrupt the task when the goniometric trace, displayed on a screen interface, exceeded two horizontal lines delineating the allowable range of motion. During remote data extraction, the exact moment when the arm angle exceeded the indicated limit was identified for analysis, with a characteristic deviation of approximately ±2 degrees inherent in the instrument.

VE was provided by the operator himself. Expressions chosen included the following: “Stay focused”, “Go for it, you’re stronger than you think”, “Keep going, you’re doing an amazing job”, “Don’t give up”, “You were born to win”, “Fatigue is temporary, satisfaction is eternal”, “Give your all”, “Believe in yourself”. These words were pronounced repeatedly to ensure that the subject was continuously encouraged throughout the test, avoiding periods of silence. The music was chosen according to the individual preferences of the participant. Both the VE and the music were presented at a level between 75 and 80 decibels to ensure that they were clearly audible without being intrusive or damaging to hearing. One week prior to the start of the study, each participant’s 1RM was estimated using the Boyd Epley multiple repetition method [35]. In addition, the participants were given detailed instructions on how to maintain their usual diet, ensure adequate hydration, avoid alcohol and caffeine intake in the three hours before the test, and abstain from physical activity in the 48 h before the assessment.

### 2.5. EMG Signal Processing

Bipolar surface electrodes were placed on the dominant side of the participant’s body, targeting three different muscle groups: the biceps brachii (BB), brachioradialis (BR), and triceps brachii (TB). The skin areas where the electrodes were placed were cleaned with alcohol and shaved if necessary. Electrodes were placed in accordance with the natural longitudinal orientation of the underlying muscle fibers, as described by De Luca [36]. The exact positions for electrode placement were determined according to the protocols established in the ‘Surface EMG for Non-Invasive Assessment of Muscles’ (SENIAM) guidelines [37]. The EMG signal was acquired using a wireless EMG device (Cometa Srl, Milan, Italy) with a first-order band-pass filter in the range 10–500 Hz and digitized at 2000 samples/s. The electrical signals produced by the muscles were processed according to specific guidelines. The signals were filtered at a specific range (20–450 Hz), then rectified, smoothed with a low-pass filter (5 Hz, 4th-order Butterworth), and finally normalized with respect to the peak activity obtained from the reference signal, thus transforming the entire signal into a relative scale where the reference peak value represents 100%.

In order to assess the level of excitation of the BB and TB muscles, the study measured the following EMG signal parameters: (i) the specific point during the test, expressed as a percentage, at which the highest level of muscle excitation occurred (peak value); (ii) the mean amplitude (mV); (iii) the trend coefficient of muscle excitation, expressed as a percentage of the time slope; and (iv) the Area Under the Curve (AUC) in units of %·s. The AUC represents the integral of the EMG signal, i.e., the total volume of electrical activity occurring during the task. In addition, (v) the co-contraction coefficient was calculated, defined as the ratio of activation of the TB muscle to activation of the BB muscle throughout the specified duration of the task.

**Figure 2:**
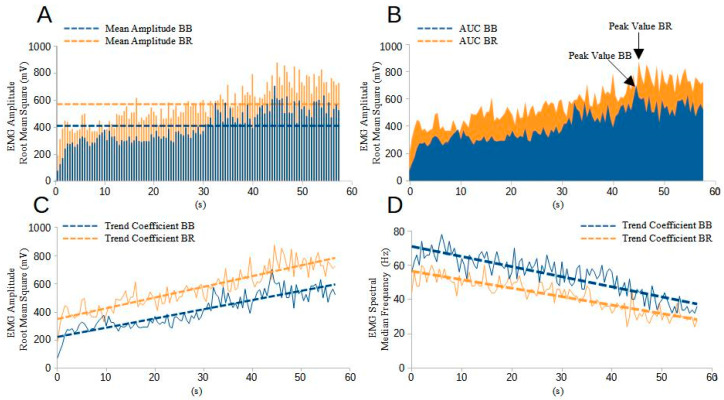
Representation of EMG outcome measures evaluated during a 57.5 s (s) task performed by a participant under verbal encouragement conditions in the biceps brachii (BB) and brachioradialis (BR) muscles. (**A**) Shows the amplitude of the EMG signal, where the solid line indicates the average amplitude measurements during the task. (**B**) Displays the area under the curve (AUC) and the point during the task when the EMG signal peak occurs. (**C**) Depicts the amplitude of the EMG signal, with the solid line indicating the trend coefficient as a measure of muscle recruitment over time. (**D**) Illustrates the myoelectric manifestations of fatigue, where the solid line represents the trend coefficient as a measure of muscle fatigue manifestations over time.

To analyze the myoelectric manifestations of fatigue, an analysis of the EMG signal spectrum was carried out using a 0.5 s time window, starting exactly at the onset of activity. The Fast Fourier Transform (FFT) was used to calculate the linear magnitude of the FFT for the selected data segments. The Median Frequency (MDF) was identified as the frequency corresponding to the 50th percentile of the cumulative energy distribution in the frequency spectrum, marking the division of the energy spectrum into two equivalent parts. The shift in the signal power spectrum towards lower frequencies indicated a reduction in the conduction velocity in the muscle fibers. This phenomenon can be attributed to changes in the intracellular pH, which tends to become more acidic due to the increased concentration of H+ ions resulting from the hydrolysis of ATP to ADP and the accumulation of lactic acid during the performance of the task [38]. The entire analysis process, including FFT and MDF determination, was facilitated using open-source Python software provided by Anaconda Inc. The resulting analysis produced a graph showing the values of MDF over time. To evaluate the temporal evolution of MDF, a linear fit of the dataset was performed, and the slope (temporal slope of MDF) was derived. These slopes were then normalized to the value of the regression line at the start time of the first activation interval, analyzed, and expressed as percentages. A gradual decrease in MDF during exercise indicates the presence of muscle fatigue [39]. In summary, in addition to measuring the task duration, four EMG outcome variables were calculated to analyze muscle activation, one variable for co-contraction, and one variable to quantify the myoelectric manifestations of fatigue. Figure 2 provides useful visual information on outcome variables through an EMG representation of an untrained participant under VE conditions.

### 2.6. Statistical Analysis

Prior to any statistical analysis, the dataset underwent a preliminary visual inspection to identify any potential outliers and ensure the integrity and accuracy of the subsequent analyses. Descriptive statistics were then performed, presenting the collected data in terms of mean and standard deviation to provide a clear, concise overview of the characteristics of the dataset. Following this initial assessment, we tested the assumptions necessary for conducting a two-way analysis of variance (ANOVA), namely the normal distribution of the continuous dependent variables and the assumption of sphericity. This was to ensure the validity of our results. The two-way ANOVA was then used to test for the presence of statistically significant differences in performance scores across the three experimental conditions (Standard, Music, VE), the training status, and their interaction, thereby testing the null hypothesis of no variance in mean scores across conditions and the training status. Only the interactions effects were hereby reported, being the findings of greater interest for the study.

As the two-way ANOVA indicated significant differences between groups, subsequent post hoc analyses were performed on the estimated marginal means using Tukey’s HSD (Honestly Significant Difference) correction. Also, for each pairwise comparison between conditions (Standard vs. Music, Standard vs. VE, and VE vs. Music), we calculated the effect size using Cohen’s d. This statistical measure provides a standardized magnitude of the difference between two group means relative to their combined standard deviation, thus providing a quantifiable measure of the significance of the effect. Commonly accepted thresholds for interpreting Cohen’s d indicate that a value of 0.20 is considered a small ES, 0.50 indicates a medium ES, and 0.80 signifies a large ES [40]. All statistical procedures were performed using the open-source software R (R version 4.2.3, The R Foundation for Statistical Computing).

## 3. Results

Based on the inclusion criteria, we assessed 30 physiatrists from the Policlinico San Martino Hospital, aged between 27 and 40 years. Out of these 30, two were excluded since they did not meet inclusion criteria. Finally, 28 physiatrists were recruited and divided into two groups according to their physical activity habits (Table 1).

Of these participants, 15 were untrained, with a mean age of 29.57 ± 2.77 years (7 females). Another 13 were trained, with a mean age of 32.92 ± 2.90 years (3 females). The trained participants showed significant differences in endurance physical activity (t = 6.43, *p* < 0.001) and muscle-strengthening activity (t = 3.03, *p* = 0.005) compared to the untrained participants.

### 3.1. Task Duration

**Figure 3:**
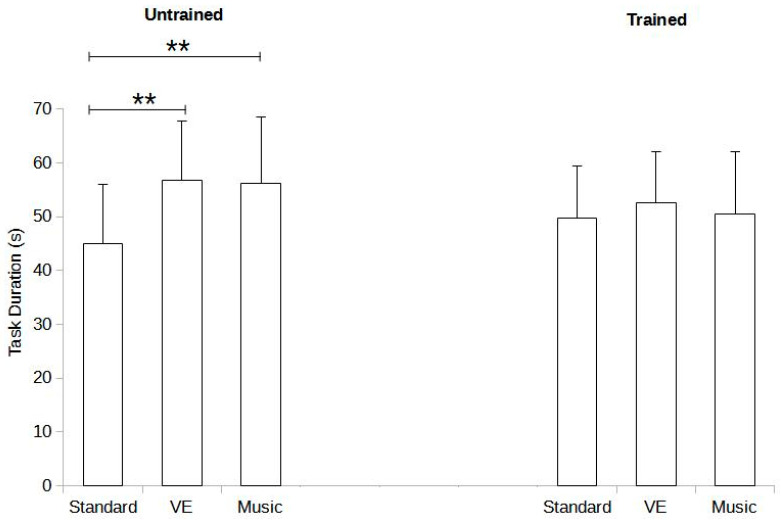
Task duration in seconds (s) as a function of training state (trained and untrained) and experimental condition (standard, verbal encouragement [VE], and music). ** indicates *p*-value < 0.01.

Significant effects were identified for the interactions between training status and the experimental condition (F = 3.693, *p* = 0.029). Post hoc analysis for the untrained group revealed significant differences between standard and music conditions (mean difference [MD] = −15.26%, *p* < 0.003, Cohen’s d = −1.396), as well as between standard and VE conditions (MD = −15.85%, *p* < 0.002, Cohen’s d = −1.450). However, no significant differences were found between music and VE conditions (MD = −0.59%, *p* = 1.000, Cohen’s d = 0.054). In contrast, the trained group showed no significant differences in the post hoc analysis (standard vs. music: MD = −0.81%, *p* = 1.000, Cohen’s d = 0.074; standard vs. VE: MD = −2.83%, *p* = 0.986, Cohen’s d = 0.259; music vs. VE: MD = −2.02%, *p* = 0.997, Cohen’s d = 0.185). Figure 3 illustrates task duration in relation to training status and experimental condition.

### 3.2. Muscle Excitation

#### 3.2.1. Mean Amplitude

**Figure 4:**
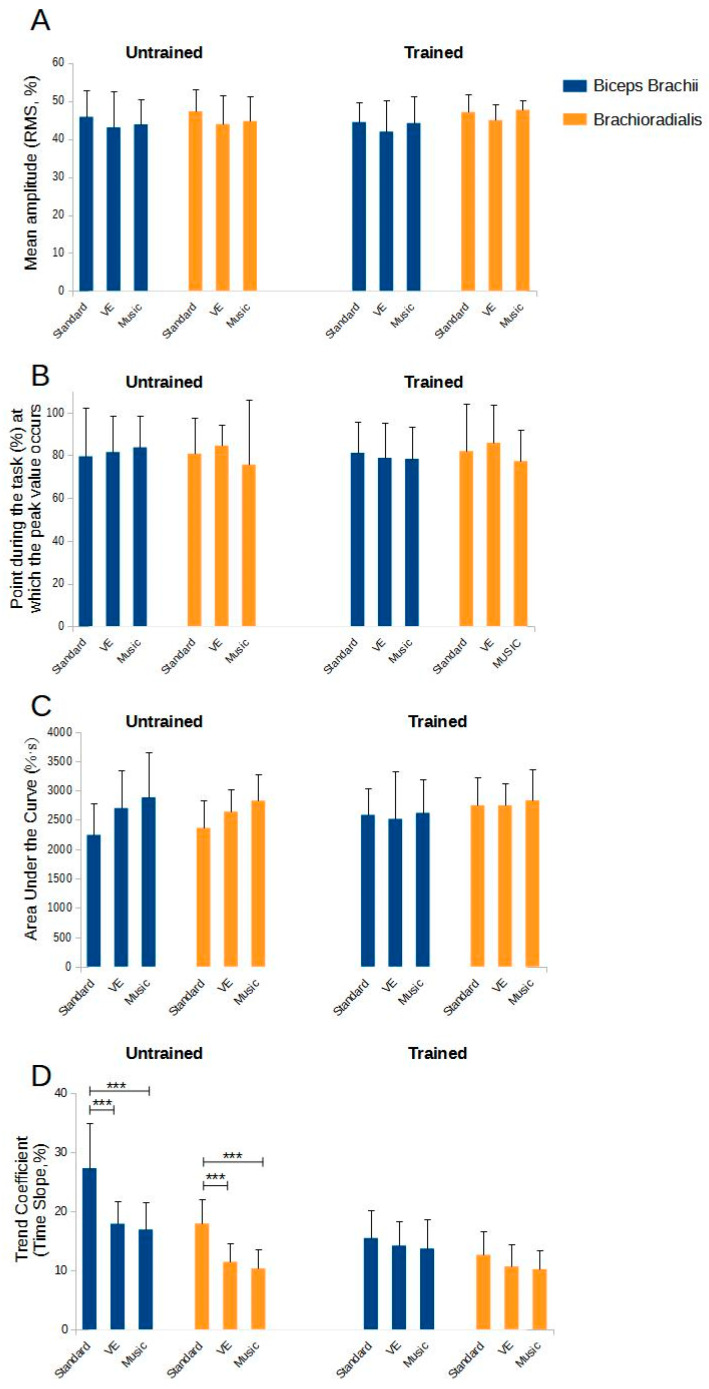
Outcome measures of muscle excitation as a function of training state (trained and untrained) and experimental condition (standard, verbal encouragement [VE], and music) in the biceps brachii and brachioradialis muscles. (**A**) shows the mean amplitude values of the EMG signal. (**B**) shows the area under the curve. (**C**) shows the point during the task when the EMG signal peak occurs. (**D**) shows the slope values of the trend coefficient of the EMG signal, which is an indicator of muscle recruitment over time. *** indicates *p*-value < 0.001.

For the BB muscle, no significant effects were observed for the interactions between training status and the experimental condition (F = 0.111, *p* = 0.895). Similarly, for the BR muscle, no significant differences were found (F = 0.111, *p* = 0.895). Figure 4A depicts the mean amplitude values relative to training status and experimental condition.

#### 3.2.2. Peak Value

No significant effects were found for the interactions between training status and the experimental condition for the BB muscle (F = 0.297, *p* = 0.744) or the BR muscle (F = 0.001, *p* = 0.999). Figure 4B depicts the peak value in relation to training status and experimental condition.

#### 3.2.3. Area under the Curve

The AUC analysis revealed no significant effects for the interactions between training status and the experimental condition for the BB muscle (F = 1.826, *p* = 0.168) or the BR muscle (F = 1.370, *p* = 0.259). Figure 4C shows the AUC values relative to training status and experimental condition.

#### 3.2.4. Time Slope of the Trend Coefficient

A significant effect was detected for the BB muscle for the interactions between training status and the experimental condition (F = 6.060, *p* = 0.004). The post hoc analysis for the untrained group showed significant differences between standard and music conditions (MD = 10.39%, *p* < 0.001, Cohen’s d = 2.009) and standard and VE conditions (MD = 9.40%, *p* < 0.001, Cohen’s d = 1.818), but not between music and VE conditions (MD = −0.99%, *p* = 0.995, Cohen’s d = 0.191). Conversely, the trained group showed no significant differences (standard vs. music: MD = 1.81%, *p* = 0.947, Cohen’s d = 0.350; standard vs. VE: MD = 1.29%, *p* = 0.988, Cohen’s d = 0.249; music vs. VE: MD = −0.56%, *p* = 1.000, Cohen’s d = 0.102).

For the BR muscle, significant effects were observed for the interactions between training status and the experimental condition (F = 4.340, *p* = 0.016). The post hoc analysis for the untrained group revealed significant differences between standard and music conditions (MD = 7.61%, *p* < 0.001, Cohen’s d = 2.135) and standard and VE conditions (MD = 6.51%, *p* < 0.001, Cohen’s d = 1.826) but not between music and VE conditions (MD = −1.10%, *p* = 0.958, Cohen’s d = 0.309). The trained group showed no significant differences (standard vs. music: MD = 2.45%, *p* = 0.501, Cohen’s d = 0.688; standard vs. VE: MD = 1.99%, *p* = 0.715, Cohen’s d = 0.557; music vs. VE: MD = −0.47%, *p* = 0.999, Cohen’s d = 0.132). Figure 4D depicts the values of the time slope of the trend coefficient in relation to the training state and the experimental condition.

#### 3.2.5. Co-Contraction Coefficient

No significant effects were found for the co-contraction coefficient concerning the interactions between training status and the experimental condition (F = 0.030, *p* = 0.970).

#### 3.2.6. Myoelectric Manifestations of Muscle Fatigue

**Figure 5:**
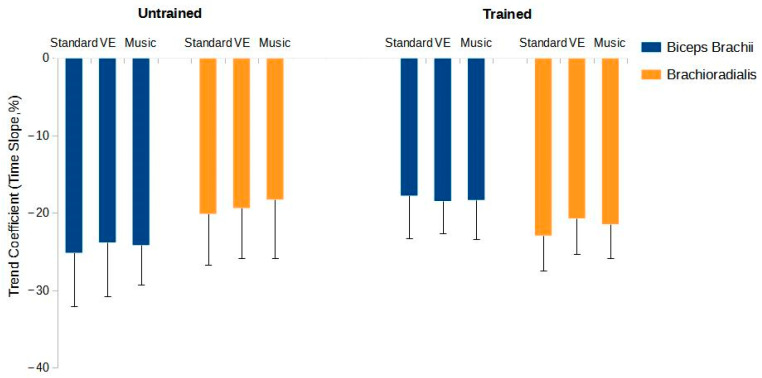
Time slope values of the trend coefficient, which is an indicator of myoelectric manifestations of fatigue over time relative to training status (trained and untrained) and experimental condition (standard, verbal encouragement [VE], and music).

For the BB muscle, no significant effects were found for the interactions between training status and the experimental condition (F = 0.239, *p* = 0.788). Similarly, no significant effects were found for the BR muscle (F = 0.190, *p* = 0.828). Figure 5 provides a visual representation of the myoelectric manifestations of muscle fatigue values relative to training status and experimental condition.

## 4. Discussion

External motivational stimuli have been shown to improve exercise performance. However, the neurophysiological mechanisms underlying this improvement remain poorly understood. This single-blind randomized crossover study investigated the effects of VE and music on task duration, muscle excitation, and the myoelectric manifestations of muscle fatigue, as assessed by using EMG, during an isometric task to exhaustion. Participants with varying levels of weekly physical activity, measured in MET minutes, were included in the study.

### 4.1. Task Duration

The untrained population increased their physical endurance, as evidenced by a longer task duration, when exposed to either VE or self-selected music. Specifically, the mean task duration without external stimuli was 45 s. This duration increased by 12 s when the task was performed with VE and by 11 s when the task was performed with music.

This finding is consistent with numerous previous studies in a variety of sports and experimental settings, although the magnitude of the improvement is smaller compared to the total task duration. For example, there were time improvements of 22 s in 10 km cycling trials [26] and 10 s in 1.5 mile running trials [25] with musical accompaniment. In the latter case, however, the substantial improvement did not reach statistical significance. Similarly, the use of VE resulted in significant reductions of 1 s in the 200 m freestyle swim [11] and 5 s in sprint tests with repeated changes in direction [41]. It is thought that the increase in self-esteem resulting from VE, together with music’s ability to distract from fatigue and increase motivation during physical activity, are the psychological underpinnings of these benefits [7,21].

No significant differences were found between the two motivational feedback conditions. This result was due to the fact that almost half of the untrained participants showed greater improvements in task duration with music (7 out of 15), while the others found VE exposure more beneficial. This trend was also found in the only study that compared the effects of VE and music on repeated sprinting with change in direction in young basketball players [31]. In particular, the authors found no significant changes in total time (sum of 10 sprints) and peak time (fastest sprint time) between the two experimental conditions. Furthermore, the authors discussed these results by highlighting how the uniqueness of everyone is clearly manifested in their preferences for receiving external feedback, underscoring that personal choice prevails over the hypothetical superiority of one condition over another.

In the analysis of trained participants, the study found minimal and statistically insignificant variations in task duration (+3 s for VE and +1 s for music, compared to the standard condition), indicating the possible presence of a ceiling effect on motivation. We suggest that trained participants may have already reached a plateau of maximum motivation, making them less receptive or susceptible to additional external motivational stimuli. These results are also consistent with existing evidence in the literature suggesting that individuals with prior training experience may have developed advanced cognitive skills [19,30]. Such skills would reduce the need to rely on external stimuli to improve performance. This implies that through experience and constant practice, these individuals have honed effective mental methods and strategies capable of improving training effectiveness independently of external motivational or distracting factors [18].

Also, in the study by Engel et al. [19], experienced adult CrossFit athletes were tested in a randomized order under two conditions, with and without VE. It was found that there were only small differences in the maximum weight lifted in the squat, with an increase of 2 kg in the presence of VE. In addition, no significant differences were observed between the two conditions in high-intensity functional training tasks, measured as the maximum number of repetitions possible. This dynamic is also supported by the study of senior judo athletes, who showed slightly lower maximal isometric grip strength in the presence of VE, suggesting a possible preference on the part of these athletes for a quieter, more focused environment that promotes concentration rather than the addition of external distractions [18].

In a parallel line of research, Argus et al. [17] observed modest improvements in mean peak power and mean peak velocity during a bench throw exercise, with increases of 1.8% and 1.3%, respectively, when rugby players received VE immediately after each attempt. The authors hypothesized that these limited improvements may be due to the ability of elite athletes to recruit a greater percentage of muscle fiber than untrained individuals. Therefore, for less experienced or untrained individuals, verbal feedback and motivational strategies may have a greater impact on muscle activation, leading to more significant improvements in performance.

Similarly, research conducted by Bigliassi et al. [42] explored the impact of listening to music on the performance and psychophysical responses of trained cyclists during a 5 km time trial. They found that the effect of music, whether listened to before or during exercise, did not lead to significant changes in performance compared to a control condition without music.

### 4.2. EMG Assessment (Muscle Excitation and Myoelectric Manifestations of Fatigue)

In trained participants, the phenomenon whereby the level of muscular excitation and myoelectric manifestations of fatigue remained stable, regardless of external stimuli, was due to the constancy of their task duration. In other words, the absence of significant variations in performance time of these participants was reflected in uniform EMG measurements across different experimental conditions. In contrast, a significant variation in task duration in response to stimuli was observed in untrained participants. However, despite these variations, the analysis carried out showed no changes across the three conditions of the mean amplitude, peak value, and AUC, indicating a similar level of neuromotor engagement, leading to a similar level of myoelectric manifestations of fatigue. Interestingly, when feedback was provided, either through VE or the use of music, there was a significant reduction in the time slope of the trend coefficient compared to the control environment without feedback for both muscles tested (time slope for BB −9% for VE and −10% for music, and time slope for BR −7% for VE and −8% for music).

The time slope of the trend coefficient measures the progressive recruitment of motor units that occurred during the task according to Henneman’s principle [43]. This principle states that during submaximal tasks that are prolonged over time, the smaller motor units, which are less susceptible to rapid fatigue, are activated first, with progressive involvement of the larger motor units depending on the intensity and duration of the task. A greater time slope of the coefficient indicates more rapid recruitment of the larger motor units than a smaller time slope. Therefore, the observed phenomenon of a more gradual recruitment of the larger and more easily fatigued motor units in the VE and musical conditions could explain the better temporal performance. In other words, the delayed activation of these motor units, which are less energy efficient but necessary to sustain intense effort, probably allowed the subjects to maintain an optimal level of effort for a longer period of time, resulting in improved performance and similar degrees of myoelectric fatigue compared to shorter tasks.

This phenomenon is in line with what is known about the psychological and neuromuscular effects of music. Music not only helps reduce the perception of fatigue through changes in attentional focus [21] but also promotes an improvement in the efficiency of muscle contractions [44]. The relationship between attention and muscular efficiency is described by the constrained action hypothesis [45]. It suggests that focusing attention on external elements, such as emotions or memories evoked by music, rather than on internal muscular sensations, may facilitate the use of more automatic control mechanisms. This approach could contribute to greater fluidity and stability of muscle contraction, promoting more gradual and efficient recruitment of motor units without the interruption of conscious monitoring of actions [46]. Conversely, focusing on one’s muscular sensations (internal attention) during a task could lead to a more conscious and deliberate control of the action, which may interfere with the efficiency of the contraction, resulting in a less stable and more fragmented execution [45,47].

Contrary to expectations based on the supposed role played by the startle mechanism [3], VE did not induce significant changes in participants’ muscle excitation, a result that seems to contradict evidence from previous studies. For example, the study by Belkhiria et al. [4] analyzed the effects of VE on maximal isometric force and EMG parameters in twenty-three participants and found a 21% increase in force production and a 12% increase in muscle excitation with VE compared to the condition without encouragement. Also, Binboğa et al. [14] found a 10% increase in muscle excitation in the sural triceps muscle group during a maximal plantar flexion contraction in participants with low conscientiousness, although the effect on motor output was not analyzed.

This discrepancy raises questions about the universal applicability of the startle mechanism, suggesting the presence of additional variables or specific contexts in which the effectiveness of VE may vary significantly. Furthermore, most studies of VE in controlled contexts focus on maximal contractions, leaving little room to explore the management of motor recruitment under more variable and less extreme conditions.

### 4.3. Limitations

This study has several limitations that need to be acknowledged. First, we calculated 1RM only once, one week before the start of the study. The 1RM is a measure that can fluctuate over time, which could lead to errors in calculating the correct weight (80% of the 1RM) for the three trials. The reason for this choice was that the untrained population involved was very sedentary, with approximately 113 MET minutes of muscle-strengthening activity. Therefore, even an exercise such as the indirect calculation of 1RM could have fatigued the participants and compromised the study. Furthermore, we believe that, although there was an inherent error in the 80% estimate of 1RM used in the tasks, randomization ensures that this error remains consistent across tasks and thus does not favor one type of task over another. Another limitation of the study is the small sample size, which according to the a priori sample size and power analysis may be slightly underpowered. This could affect the generalizability of the results, making it difficult to draw broader conclusions and highlighting the need for further research with larger and more diverse populations to validate our findings.

## 5. Conclusions

The study investigated the effects of VE and music on physical performance during an isometric exhaustion task and found an increase in task duration in untrained participants compared to the condition without additional stimuli. Furthermore, the use of external stimuli in these participants led to a decrease in the time slope of the trend coefficient in the muscles studied (BB and BR), suggesting a more homogeneous and efficient muscle recruitment process. This dynamic may have enabled the subjects to maintain a sustained effort for longer periods of time, resulting in increased temporal performance and myoelectric responses to muscle fatigue comparable to that recorded during exercises of shorter duration (without external stimuli). Comparison of the effects of music and VE showed no significant differences, suggesting that preference for one or the other motivational tool is a matter of personal taste rather than superiority in terms of effectiveness. On the contrary, in the trained participants, no significant differences were found between the experimental conditions, either in the duration of the task or in the variables related to muscle excitation and myoelectric manifestations of fatigue (EMG assessment), suggesting the presence of a ceiling effect of motivation. These findings highlight the critical role of external stimuli in improving motor task performance and explains the underlying neurophysiological mechanisms in untrained participants. They also suggest a potential application in physical rehabilitation programs to increase patient engagement and maximize the benefits of therapeutic exercise, particularly for individuals with low motivation or a tendency toward early fatigue. For trained participants, the lack of effects should temper the strong belief that motivational feedback is truly beneficial, potentially prohibiting its use in certain competitive contexts.

## Figures and Tables

**Table 1:** Mean and standard deviation of the demographic and anthropometric characteristics of female and male participants, weekly endurance physical activity (Endurance), and muscle-strengthening activity (Strength) expressed in MET minutes per week, as well as the weight used during the task (Dumbbell, kg).

Participant	Sex	Age (Year)	Body Mass (Kg)	Height (cm)	Endurance (MET)	Strength (MET)	Dumbbell (Kg)
All	10f 18m	31.19 (3.58)	71.15 (11.88)	174.70 (8.77)	943.89 (635.84)	495.21 (551.40)	11.63 (3.71)
Untrained	7f 8m	29.57 (2.77)	69.71 (12.79)	174.36 (8.84)	441.33 (194.67)	112.67 (36.78)	9.57 (2.28)
Trained	3f 10m	32.92 (2.90)	72.69 (11.91)	175.08 (9.04)	1523.77 (430.65)	936.62 (536.14)	13.92 (3.68)

Abbreviations: f = female; m = male; MET = Metabolic Equivalent of Task.

## Data Availability

Data from this study are available from the corresponding author upon reasonable request.
